# Digital Storytelling Intervention to Promote Human Papillomavirus Vaccination Among At-Risk Asian Immigrant Populations: Pilot Intervention Study

**DOI:** 10.2196/46951

**Published:** 2023-10-04

**Authors:** Angela Chia-Chen Chen, Sunny Wonsun Kim, Lihong Ou, Michael Todd, Linda Larkey

**Affiliations:** 1 College of Nursing Michigan State University East Lansing, MI United States; 2 Edson College of Nursing & Health Innovation Arizona State University Phoenix, AZ United States

**Keywords:** digital storytelling, human papillomavirus, immigrants, Korean, Vietnamese, vaccination

## Abstract

**Background:**

The high morbidity, mortality, and economic burden attributed to cancer-causing human papillomavirus (HPV) calls for researchers to address this public health concern through HPV vaccination. Despite disparities in HPV-associated cancers in Korean Americans and Vietnamese Americans, their vaccination rates remain low. Evidence points to the importance of developing culturally and linguistically congruent interventions to improve HPV vaccination rates. Digital storytelling (a specific form of cultural narrative) shows promise as an effective culture-centric health promotion strategy.

**Objective:**

The aim of this quasi-experimental single-group study was to assess the feasibility, acceptability, and preliminary effects of a culturally and linguistically congruent digital storytelling intervention on Korean American and Vietnamese American mothers’ attitudes and intention in vaccinating their children against HPV. We also examined if the association between attitudes and intention differed by their child’s sex (boy vs girl) and by ethnicity (Korean American vs Vietnamese American).

**Methods:**

Participants were recruited via multiple avenues (eg, ethnic minority community organizations, social media, and flyers posted in local Asian supermarkets and nail salons). Web-based, valid, and reliable measures were administered to collect data preintervention and postintervention. Descriptive statistics, paired and independent sample *t* tests, the chi-square test, and the McNemar test were used to describe the distributions of variables and to examine the differences between subgroups and changes in key variables over time. Logistic regression models were used to examine associations of mothers’ HPV- and vaccine-related attitudes with vaccination intention and to explore if the association between attitudes and vaccination intention differed by the target children’s sex or ethnicity.

**Results:**

In our sample of 50 Korean American mothers (mean age 42.8, SD 4.8 years) and 114 Vietnamese American mothers (mean age 41.5, SD 5.4 years), 36% (18/50) of Korean American and 51% (58/114) of Vietnamese American mothers reported that their children received free or reduced-price lunches at school. After the intervention, mothers’ attitudes toward HPV and the vaccine (*t*_163_=2.49, *P*=.01) and intention to vaccinate their children improved significantly (*X*^2^_1_=18.38, *P*<.001). The measure of mothers’ negative attitudes toward HPV and the vaccine was significantly associated with higher vaccination intention (odds ratio 0.27, 95% CI 0.14-0.51; *P*<.001), adjusting for background variables (sociodemographic characteristics) and other HPV-related variables (family cancer history, prior HPV education, and HPV communication with health care providers). Findings did not suggest that a child’s sex or ethnicity moderated the association between attitudes and vaccination intention.

**Conclusions:**

This remotely delivered intervention using digital stories was feasible and acceptable, and showed preliminary effects on promoting Korean American and Vietnamese American mothers’ intention to vaccinate their children against HPV. Future research that uses a randomized controlled trial design with a larger and more diverse sample and includes children’s vaccination status will help understand the effect of the intervention.

## Introduction

The human papillomavirus (HPV) is the most common sexually transmitted infection in the United States; more than 42 million Americans are infected with types that cause diseases and 13 million of them, including adolescents, are infected each year [1]. HPV infection causes cancers in both men and women: it is estimated to be associated with >90% of anal and cervical cancers, 70% of vaginal and vulvar cancers, 60%-70% of oropharynx cancers, and 60% of penile cancers [2]. Annual deaths attributed to HPV-related cancers have been estimated to be over 7000 with a substantial economic burden (US $3.8 billion) in the United States [3]. Vigorous prevention efforts including HPV vaccination for boys and girls at 11 or 12 years of age are recommended by the US Centers for Disease Control and Prevention [4], but uptake remains low in many population groups. While cervical cancer rates may be decreasing, anal and oropharynx cancers show an increase, causing concern about gender differences in HPV vaccination rates [5].

Asian American women are disproportionately affected by cervical cancer. Among Asian American subgroups, Vietnamese American women (18.9 per 100,000) and Korean American women (11.9 per 100,000) had the highest rates of cervical cancer mortality compared with non-Hispanic White women (7.1 per 100,000) [6,7]. These findings are concerning because cervical cancer is preventable with HPV vaccination and Papanicolaou test (Pap smear) cancer screening [8]. Moreover, vaccination uptake rates were low across ethnic groups in the United States [9]. For example, Asian American adolescent girls aged 9-17 years had significantly lower HPV vaccine initiation (12.4% vs 27.2%) and completion (1.9% vs 10.3%) rates compared with all other races or ethnicities combined [10]. For Korean American and Vietnamese American women across age groups, the HPV vaccine completion rate is 33.3% (53/160) for Korean American women [11] and 9% (11/113) for Vietnamese American immigrant women [7]. These rates, however, are substantially below the 80% goal targeted by Healthy People 2030 [12]. While there are gender differences in vaccination rates nationally [13], currently there are no data available for Asian American men on HPV vaccination rates or cancers. This concerning lack of data highlights a need to focus intervention efforts on both boys and girls.

Given the need for parental consent and the unique context of mothers’ health-related decision-making in Asian culture, it is crucial to engage Korean American and Vietnamese American mothers in HPV vaccination [7,14-16]. Mothers play an important role in HPV vaccination uptake in their children, with mothers as the primary attendee at doctors’ visits, especially in Asian populations [16]. Factors such as mothers’ limited English proficiency and lack of knowledge about the HPV vaccine, or beliefs that vaccination would encourage children’s premarital sex have significantly contributed to the lower rates of HPV vaccination in the Korean American [14,15] and Vietnamese American [17] populations. In particular, rates are lower in children of first-generation Korean American and Vietnamese American immigrant mothers (defined as individuals who were born outside of the United States) [18].

To date, the existing limited literature has focused on older age groups and other Asian American ethnic groups. In a pilot randomized controlled trial (RCT) examining the feasibility, acceptability, and preliminary efficacy of a web-based storytelling intervention to promote HPV vaccination in 104 female Korean American college students aged 18-26 years [19], participants in the intervention group demonstrated better knowledge and higher vaccination than the control group at the 2-month follow-up. Lee et al [20] conducted an RCT to examine the feasibility and effectiveness of a storytelling intervention among Khmer American mother-daughter dyads on HPV vaccination behaviors. The acceptability of the intervention was high, and vaccination intent increased. Other research [14] found that informing Korean American mothers about HPV can increase their HPV knowledge. However, other key variables, such as vaccination intention and behavior, were not measured. While Korean American and Vietnamese American mothers’ attitudes, beliefs, and intentions to have their children vaccinated are key to increased HPV vaccination [14,15,17], there is a paucity of rigorous research engaging Korean American and Vietnamese American mothers in the process of developing culturally and linguistically congruent interventions. Furthermore, there are few studies focused on both boys and girls, despite vaccination being effective in preventing HPV-associated cancers in both sexes.

Digital storytelling (DST), which combines oral storytelling with computer technology, has been used as a tool to communicate culturally relevant messages in health, education, and community settings [21-23]. The process of DST, an innovative community-based participatory research method, involves the production of individuals’ own brief visual stories presented in their own voice, incorporating photographic images, music, and artwork of their choice in a workshop setting guided by professionals, these stories are 1-3 minutes in length [22,23]. Research examining DST interventions has demonstrated its potential benefits for promoting different health behaviors including HPV vaccination among youth and adult participants [17,19]. In our pilot studies, we have developed Korean American and Vietnamese American mothers’ personal stories related to HPV and vaccination, the experience of creating and talking about their HPV experiences [24]. Guided by a model of the effects of narrative for culture-centric health promotion [25], we suggest that culturally resonant or embedded stories generate the experience of transportation (emotional engagement and getting “carried away” by the story) and identification (with characters, story, and cultural elements). These experiences, combined with story content demonstrating or promoting a particular behavior serve to change mothers’ attitudes about the behavior, which in turn, drive their intention to perform a specific behavior. DST has not been rigorously tested, however, in addressing Korean American and Vietnamese American mothers’ barriers to vaccinating their boys and girls against HPV, despite the potential of this low-cost and accessible communication mode to be tailored to mothers’ concerns in a culturally and linguistically appropriate way. In addition, little is known about how the process of viewing and engaging a personal story told by other Korean American and Vietnamese American mothers may be linked to the unique cultural view of the HPV attitudes, intentions, and behaviors to promote vaccination through vicarious social modeling.

This pilot study assessed the feasibility, acceptability, and preliminary effect of an innovative, remotely delivered, and culturally and linguistically congruent DST intervention consisting of stories of personal lived experiences to improve Korean American and Vietnamese American mothers’ attitudes and intentions in their children’s HPV vaccination. This research addressed gaps in the prior research by targeting 2 separate Asian American subgroups: Korean Americans and Vietnamese Americans, both of which have disproportionate rates of HPV-associated cervical cancer and low vaccination rates. Our findings also expand upon prior research by examining if the intervention effects differ by the child’s sex (boy vs girl) and by ethnicity (Korean American vs Vietnamese American).

## Methods

### Design and Sample

We used a quasi-experimental single-group pretest-posttest design to examine the preliminary effect of the DST intervention on Korean American and Vietnamese American mothers’ attitudes toward HPV vaccination and their intentions to have their child vaccinated. Adult women (aged 18 years or older) were recruited if they (1) self-identified as Korean American or Vietnamese American or as Korean or Vietnamese immigrants, (2) were first-generation immigrants born outside of the United States, and (3) had 1 or more children aged 9-14 years old who had not been vaccinated against HPV. English fluency was not required, given the multilingual (English, Korean, Vietnamese) features of the intervention and the multicultural and multilingual resources of our team. If an eligible mother had more than 1 child aged 9-14 years old who had not received the HPV vaccination, we asked her to answer questions based on the oldest child. The sample included 50 Korean American and 114 Vietnamese American mothers who met the inclusion criteria.

The planned sample size for this study was n=50 Korean American mothers and n=50 Vietnamese American mothers (N=100). This sample size was selected to allow for thorough evaluation of study recruitment, testing of intervention materials and procedures, participant tracking, follow-up data collection within each ethnic group, and to yield estimates of variability (or uncertainty) suitable for planning a full-scale RCT assuming small effect sizes [26,27]. Though formal hypothesis testing was not the primary study aim, power analyses were conducted prior to the study. The planned sample size was expected to afford a power of 0.80 to detect moderate within-ethnic group effects (Cohen *d*≥0.41) on continuous variables at α=.05. Due to an unexpectedly enthusiastic response from the Vietnamese American community recruited by our bilingual team members, community partners, and social media, our sample of Vietnamese American mothers was much larger than initially planned.

### Recruitment, Setting, and Procedures

We partnered with community partners (eg, Asian Pacific Community in Action and The Arizona Partnership for Immunization) and bilingual research assistants to recruit Korean American and Vietnamese American mothers via word of mouth, ethnic minority community organizations, social media (Facebook, radio broadcast, and newspaper), flyers posted in Asian supermarkets and nail salons in the state of Arizona, and web-based ethnic communities (eg, MissyUSA); these are strategies that have proven to be effective in prior research [17, 24]. Interested individuals were screened by a web-based screening survey or phone; eligible individuals were invited to meet with a trained bilingual research assistant via Zoom (Zoom Video Communications, Inc) meetings to explain the study’s purpose, procedures, and confidentiality issues. Participants received a survey link to informed consent and a baseline (T0) assessment documents. Once they consented and filled out the T0 assessment, they were led to view the digital stories. After reviewing the stories, participants were invited to complete a postintervention survey (T1) immediately. Given the safety concerns during the COVID-19 pandemic, we implemented a web-based intervention and data collection via REDCap [28].

### Intervention

This DST intervention includes 8 digital stories; each participant received brief stories (each about 3 minutes long) specifically for her ethnic group at once (ie, Korean American participants received stories developed by Korean American mothers and Vietnamese American participants received stories developed by Vietnamese American mothers). These digital stories were codeveloped with Korean American and Vietnamese American mothers who had children vaccinated against HPV through web-based DST workshops conducted between July 2021 and January 2022 [24]. These stories reflected the mothers’ rich personal and cultural experiences, attitudes, and perceptions about their children’s HPV vaccination. The content of stories represents a number of perceived barriers that an immigrant might face, including language barriers, the complexity of the health care system, competing family values, cultural perceptions about HPV (eg, talking about sexuality as a taboo topic), and lack of culturally relevant information and resources, as well as strategies for overcoming these barriers. The content also includes reasons and factors for choosing HPV vaccination for their children, such as protection for both sons and daughters, the importance of having children vaccinated against HPV-related cancers while they are young, the importance of providers’ recommendations, and family history of cervical cancer. Mothers watched the digital stories by themselves at a time and location convenient to them and were encouraged to contact the research team for any questions regarding the intervention or content and study procedures.

### Ethics Approval

The study was approved by the institutional review boards of Arizona State University (IRB#: STUDY00011207 and STUDY00011733) and all materials were carried out in accordance with relevant guidelines and regulations and translated and back-translated to Korean and Vietnamese using institutional review board protocols. Informed consent was obtained from all participants.

### Measures

We had translated study materials into Korean and Vietnamese prior to conducting the study [29]. Many of the survey items (eg, HPV attitudes) were developed while working with the target populations; these items have been tested and validated in our prior work [17,30]. In the baseline (T0) assessment, participants were asked about sociodemographic characteristics, questions about family cancer history, attitudes toward HPV and the vaccine, and other HPV-related questions. With the exception of questions about sociodemographic characteristics and health history, all questions were administered again immediately postintervention (T1).

### Outcome Measures: Feasibility and Acceptability

Feasibility was assessed based on participation rate (percentage of eligible individuals agreeing to participate) and retention (proportion retained through follow-up). Acceptability was assessed based on the proportion of participants who would recommend the intervention to others. We used the following benchmarks to determine study feasibility: 80% participation rate, 80% retention rate, and 80% of mothers recommending the intervention to others.

### Dependent Variable: Intention to Vaccinate Child against HPV

This dependent variable was measured by a binary (yes or no) question to assess mothers’ intention to vaccinate their children.

### Independent Variable: Mothers’ Negative Attitudes Toward HPV Vaccination

Mothers’ attitudes toward HPV vaccination were assessed by a 6-item measure, with item response options ranging from 1 (strongly disagree) to 5 (strongly agree). A composite score was computed as a mean of the item scores, with a potential range of 1 to 5, with higher scores indicating more negative attitudes toward HPV vaccination. Reliability in this sample was acceptable (Cronbach α=.73 and .68 at T0 and T1, respectively).

### Other Variables

Sociodemographic characteristics and health-related questions included the mother’s age, birthplace, immigration- and language-related questions, education (less than a bachelor’s degree vs a bachelor’s degree or higher), employment, if child receives free or reduced-price lunch at school, health insurance coverage, family cancer history, prior HPV education, and communication with health care providers about HPV and the vaccine. Because many participants in our prior research did not know or were hesitant to provide information about their household income, we used free or reduced-price lunch at school as an indicator of the participant’s socioeconomic status.

### Statistical Analysis

We first conducted univariate analyses (eg, of means and frequencies) to describe distributions of key variables. We used 2-tailed independent group *t* tests and chi-square tests to evaluate differences in key variables between subgroups (Korean American vs Vietnamese American). To examine changes in negative vaccination-related attitudes and in vaccination intention from preintervention to postintervention for each subgroup separately, we conducted 2-tailed paired-sample *t* tests (for attitudes) and the McNemar test (for intention). We also explored potential between-ethnic group differences in preintervention to postintervention change in attitudes and rates of intention to vaccinate as estimated by an ethnicity (Korean American vs Vietnamese American) × time (preintervention vs postintervention) interaction term in generalized linear mixed models. The model for attitudes used an identity link and Gaussian (normal) error distribution. The model for intention used a logit link and binomial error distribution. Both models included fixed effects for ethnicity, time, and ethnicity × time, along with a random participant-level intercept effect.

To examine the association between mothers’ HPV-related attitudes and mothers’ vaccination intention, adjusting for background covariates, and other HPV-related variables (family cancer history, prior HPV education, and communication with health care providers), we estimated a set of 3 hierarchical logistic regression models with vaccination intention as the outcome. Model 1 included only background variables as covariates (the mother’s ethnicity, education, and health insurance coverage; the child’s sex; and whether or not the child received a free or reduced-price school lunch). Model 2 included family cancer history, prior HPV education, and communication with health care providers along with background covariates from model 1. Model 3 included the mother’s negative attitudes toward HPV vaccination along with all covariates included in model 2. We tested the significance of individual model coefficients using 2-tailed Wald tests and improvement in model fit at each step by comparing model log likelihood values.

We also explored the potential moderating effect of the target child’s sex (boy vs girl) and ethnicity (Korean American vs Vietnamese American) on the association between the mother’s negative attitudes and vaccination intention using 2 separate logistic regression models, each with the main effects of the moderator of interest (either child’s sex or ethnicity) and the negative attitudes composite measure along with their interaction. No other covariates were included in these models.

## Results

### Feasibility and Acceptability

**Figure 1:**
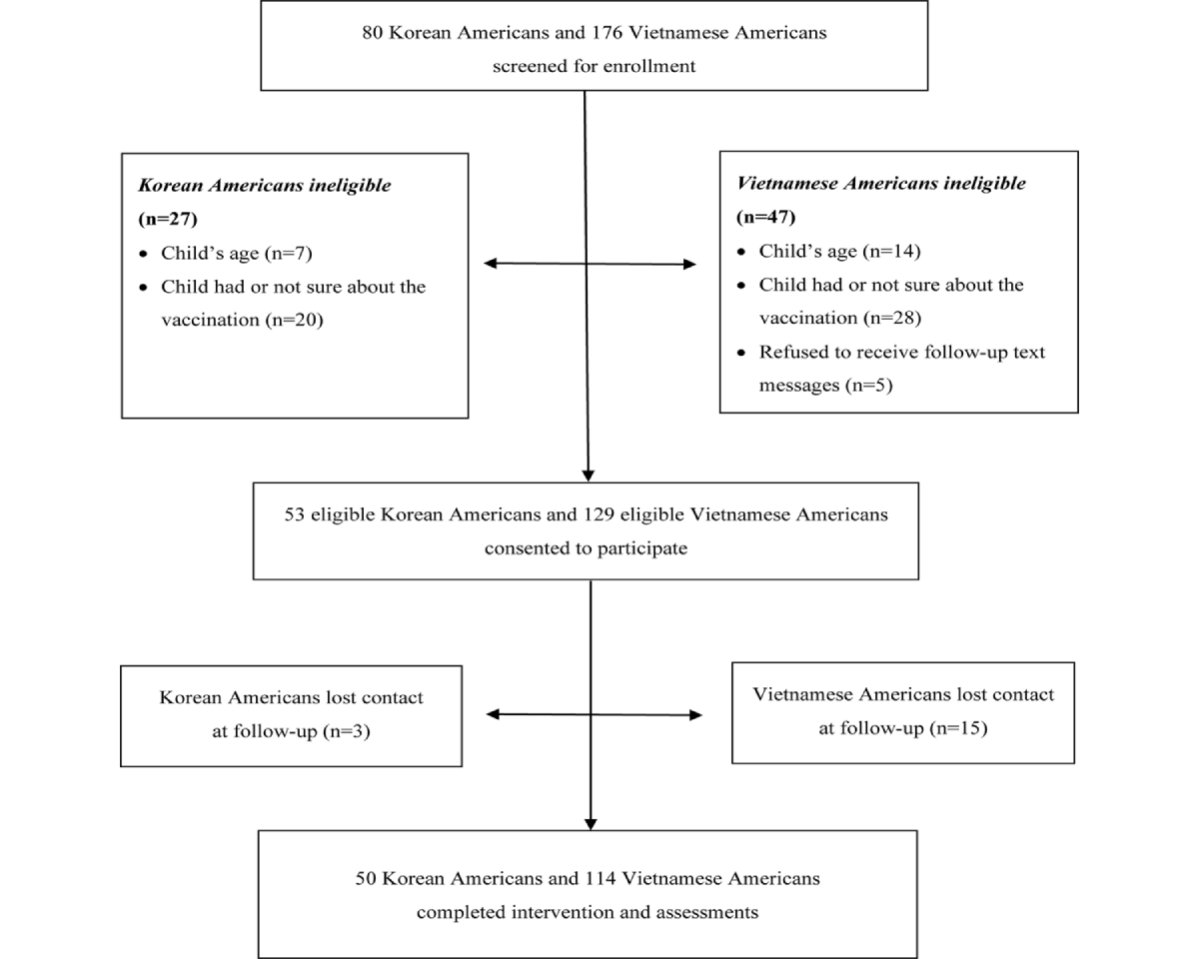
Flow chart of study participants.

This study was feasible, as evident by the 100% (182/182) participation rate for both Korean American and Vietnamese American groups, and high retention rates (50/53, 94.3% for Korean Americans and 114/129, 88.4% for Vietnamese Americans). Acceptability of the intervention was also high with 84% (42/50) of Korean American mothers and 86.8% (99/114) of Vietnamese American mothers reporting that they would recommend it to others. The numbers of individuals who screened, were eligible, and completed the intervention and assessments are presented in Figure 1.

### Sample Characteristics

The sample included 50 Korean American mothers (mean age 42.8 years, SD 4.8) and 114 Vietnamese American mothers (mean age 41.5 years, SD 5.4). The mean length since immigration was 17.4 years (SD 11.3) for Korean American mothers and 26.2 years (SD 9.1) for Vietnamese American mothers. While the vast majority (93.9%, 46/49) of Korean American mothers had a bachelor’s degree or above, less than a third (27.2%, 31/114) of the Vietnamese American mothers reported having at least a bachelor’s degree. Regarding economic situation, 36% (18/50) of Korean American mothers and 51% (58/114) Vietnamese American mothers reported children receiving free or reduced-price lunches at school, suggesting more than one-third of the Korean Americans and about half of Vietnamese Americans came from economically disadvantaged families.

Regarding cancer history, 8% (4/50) of Korean American mothers and 12% (14/114) of Vietnamese American mothers had a cancer diagnosis; 46% (23/50) of Korean American mothers and 37% (42/114) of Vietnamese American mothers reported a family history of cancer. About half (51%, 25/49) of Korean American mothers and 60% (68/114) of Vietnamese American mothers reported learning about HPV and the vaccine, and 60% (15/25) of Korean American mothers and 51% (35/68) of Vietnamese American mothers indicated that a health care provider was the main source of information. Table 1 summarizes the sample characteristics, including the target children’s age and sex reported by the mothers.

**Table 1:** Characteristics of mother participants (N=164).

Variable			Korean American (n=50)	Vietnamese American (n=114)	Total
**Children’s characteristics (n=162)**					
	Mean age of target children^a,b^ (years), mean (SD)		10.6 (1.8)	12.7 (1)	12.1 (1.6)
	**Sex of target children, n (%)**				
		Male	19 (39.6)	51 (44.7)	70 (43.2)
		Female	29 (60.4)	63 (55.3)	92 (56.8)
**Mothers’ characteristics**					
	**Education level (n=163), n (%)**				
		Lower than bachelor’s degree	3 (6.1)	83 (72.8)	86 (52.8)
		Bachelor’s degree or higher	46 (93.9)	31 (27.2)	77 (47.2)
	**Primary language spoken with children (n=164), n (%)**				
		Korean or Vietnamese	46 (92)	19 (16.7)	65 (39.6)
		English	4 (8)	93 (81.6)	97 (59.1)
		Other	0 (0)	2 (1.8)	2 (1.2)
	**Health insurance (n=164), n (%)**				
		Yes	47 (94)	89 (78.1)	136 (82.9)
		No	3 (6)	25 (21.9)	28 (17.1)
	**Working status (n=163), n (%)**				
		Yes	27 (55.1)	48 (42.1)	75 (46)
		No	22 (44.9)	66 (57.9)	88 (54)
	**Family history of cancer (n=164), n (%)**				
		Yes	23 (46)	42 (36.8)	65 (39.6)
		No	27 (54)	72 (63.2)	99 (60.4)
	**Mother’s cancer history** **(n=164), n (%)**				
		Yes	4 (8)	14 (12.3)	18 (11)
		No	46 (92)	100 (87.7)	146 (89)
	**If health care provider has discussed HPV^c^ (n=164), n (%)**				
		Yes	10 (20)	71 (62.3)	81 (49.4)
		No	40 (80)	43 (37.7)	83 (50.6)
	**Prior HPV education (n=163), n (%)**				
		Yes	25 (51)	68 (59.6)	93 (57.1)
		No	24 (49)	46 (40.4)	70 (42.9)

^a^Target children were defined as the oldest unvaccinated child aged 9-14 years in the family.

^b^Range 9-14 years.

^c^HPV: human papillomavirus.

### Mothers’ Attitudes and Intention to Vaccinate Their Children

In Table 2, summary statistics for preintervention and postintervention scores for each attitude item, the total negative attitudes composite score, and vaccination intention are presented separately by ethnic group and for the full sample.

**Table 2:** Descriptive statistics and comparisons of pretest-posttest differences on key measures (N=164).

Variable		Korean American		Vietnamese American		Total		*P* value for pre-post change
		Pretest	Posttest	Pretest	Posttest	Pretest	Posttest	
**Attitude item (n=164), mean score^a^ (SD)**								
	It is not important to have the HPV vaccination for my child because everyone will eventually die of something anyway.	1.6 (0.6)	1.4 (0.6)	1.9 (1.1)	1.9 (1.2)	1.8 (1)	1.7 (1.1)	.24^b^
	It is not necessary to have the HPV vaccination for my child because it is in God’s hands anyway.	2.12 (1)	1.67 (1)	2.29 (1.3)	2.18 (1.2)	2.24 (1.2)	2.03 (1.1)	.02^b^
	If nothing is physically wrong, then my child does not need the vaccines.	2.02 (1.1)	1.41 (0.8)	2.32 (1.4)	2.07 (1.2)	2.23 (1.3)	1.87 (1.1)	<.001^b^
	It is our obligations to protect girls, so only girls should be vaccinated.	1.52 (0.7)	1.30 (0.6)	2.39 (1.4)	2.75 (1.4)	2.14 (1.3)	2.32 (1.4)	.09^b^
	Girls won’t have kids if they are vaccinated.	3.98 (0.6)	4.08 (1)	2.25 (1.2)	2.05 (1.1)	2.78 (1.3)	2.67 (1.4)	.20^b^
	It’s shameful to have my child vaccinated because people may think they’re already sexually active at this young age.	1.40 (0.6)	1.17 (0.4)	2.31 (1.4)	1.99 (1.1)	2.05 (1.3)	1.76 (1)	.001^b^
Attitude composite score^a^ (n=164), mean (SD)		2.12 (0.4)	1.84 (0.4)	2.24 (0.9)	2.16 (0.8)	2.21 (0.8)	2.06 (0.7)	.01^b^
Vaccination intention and (%) yes responses (n=164), n (%)		43 (86)	45 (90)	60 (52.6)	84 (73.7)	103 (62.8)	128 (79)	<.001^c^

^a^Scale range 1-5. Higher scores on the attitude composite indicate more negative attitudes toward HPV vaccination.

^b^Paired-sample *t* test (*df*=163).

^c^McNemar test for paired proportions; n=164 pairs.

### Change in Attitudes Toward HPV and the HPV Vaccine

In the full sample, mothers’ negative attitudes toward HPV vaccination decreased significantly from preintervention to postintervention (pretest mean 2.21; posttest mean 2.06), t_163_=2.49, *P*=.01. The average raw (unadjusted) change in attitudes from preintervention to postintervention was larger for Korean American mothers (mean 0.28-point decrease) than for Vietnamese American mothers (mean 0.09-point decrease), but the test of the ethnicity × time interaction for this outcome was not statistically significant (Wald *X*^2^_1_=2.72, *P*=.10).

### Change in Intention to Vaccinate Child

The mothers’ intention to vaccinate their children increased significantly from 62.8% (103/164) to 78.7% (129/164) after the intervention (N=164; *X*^2^_1_=18.38, *P*<.001; exact test *P*<.001). Though the increase in the rate of intention to vaccinate for Vietnamese American mothers (60/114, 52.6% to 84/114, 73.7%) was larger than that for Korean American mothers (43/50, 86% to 45/50, 90%), the ethnicity × time interaction was not statistically significant (Wald *X*^2^_1_=1.92; *P*=.17).

### Logistic Regression: Predicting Mothers’ Intention to Vaccinate Their Children

The findings from the hierarchical logistic regression are summarized in Table 3. In neither model 1 (sociodemographic variables only) nor model 2 (sociodemographic variables plus cancer history, prior HPV education, and HPV-related communication with health care providers) was any individual covariate significantly associated with intention to vaccinate when adjusting for other covariates in the model. Both sets of covariates, however, resulted in improved model fit. In model 3 (the final model), the composite measure of mothers’ negative attitudes toward HPV and the vaccine was significantly associated with vaccination intention, such that more negative attitudes (ie, higher composite scores) were associated with a lower likelihood of intending to have the child vaccinated (odds ratio [OR] 0.27, 95% CI 0.14-0.51; *P*<.001).

Exploratory analyses did not reveal a significant interaction between either the target child’s sex (boy vs girl) or ethnicity (Korean American vs Vietnamese American) and mothers’ negative attitudes toward HPV vaccination in predicting T1 vaccination intention (OR 0.99, 95% CI 0.29-3.34; *P*=.99 and OR 6.18, 95% CI 0.48-78.90; *P*=.16, respectively).

**Table 3:** Hierarchical logistic regression estimates for prediction of mothers’ postintervention vaccination intention from postintervention negative human papilloma virus vaccination attitudes (N=161).

Predictors		Model 1^a^, OR^b^ (95% CI)	Model 2^a^, OR (95% CI)	Model 3^a^, OR (95% CI)
**Demographic characteristics**				
	Ethnicity	0.41 (0.11-1.49)	0.23* (0.06-0.92)	0.46 (0.10-2.13)
	Target child’s sex	0.93 (0.42-2.06)	0.81 (0.35-1.86)	0.88 (0.35-2.21)
	Highest education	1.71 (0.61-4.79)	1.30 (0.44-3.89)	1.37 (0.39-4.75)
	Receiving free or reduced-priced lunch	0.76 (0.34-1.69)	0.69 (0.29-1.61)	0.66 (0.26-1.70)
	Having health insurance or coverage	1.50 (0.58-3.88)	1.39 (0.50-3.87)	1.46 (0.48-4.47)
**HPV^c^-related factors**				
	Family cancer history	N/A^d^	0.79 (0.34-1.85)	0.66 (0.26-1.68)
	Prior HPV education	N/A	2.36 (0.98-5.72)	2.00 (0.75-5.32)
	Discussions about HPV with health care provider	N/A	1.95 (0.74-5.13)	1.20 (0.41-3.51)
	Negative attitudes toward HPV vaccination	N/A	N/A	0.27*** (0.14-0.51)

^a^Δ–2 log likelihood: *X*^2^_5_=10.25, *P*=.07 (model 1 vs null model); *X*^2^_3_=9.01, *P*=.03 (model 2 vs model 1); *X*^2^_1_=20.35, *P*<.001 (model 3 vs model 2).

^b^OR: odds ratio.

^c^HPV: human papilloma virus.

^d^N/A: not applicable.

*P<.05.

***P<.001.

## Discussion

### Principal Findings

In this study, we sought to address a highly significant public health problem, HPV-related cancers among at-risk and understudied Korean American and Vietnamese American populations. We examined the feasibility, acceptability, and preliminary effects of the DST intervention on vaccination attitudes and intention among a community sample of 50 Korean American and 114 Vietnamese American mothers of unvaccinated children. Although prior research examining DST interventions has demonstrated their potential benefits for promoting a variety of health behaviors among youth and adult participants [21,31], limited research has used this type of low-cost, scalable, and accessible approach, which can be tailored to culturally and linguistically appropriate ways to match mothers’ concerns, to address the low HPV vaccination rates in Korean American and Vietnamese American adolescent boys and girls.

Consistent with the growing body of literature on the effects of culturally appropriate interventions in promoting HPV vaccination among underserved populations [17,19,30,32], the increase in mothers’ intention to vaccinate their children provided preliminary support for the effectiveness of using digital stories as an intervention to promote HPV vaccination in our target populations. The success of the intervention may be explained by the unique characteristics of the intervention, which was codeveloped with Korean American and Vietnamese American mothers who shared similar cultural norms and values with our target populations. Similar to Larkey et al [33-35], Korean American and Vietnamese American mothers reported that the digital stories were highly engaging and held their attention. In line with our prior research with Korean American and Vietnamese American immigrant mothers [17,24,30], the shared experience of acculturation and motherhood, and the responsibilities of taking care of their children demonstrated in the intervention may induce emotional resonance and enhance the sense of mutual understanding. Watching stories in their native languages and having subtitles may effectively address the potential language gaps and facilitate mothers’ active engagement with stories and characters. For example, some participants in our study expressed appreciation that their native language was used in stories, as their limited English proficiency would have made it more difficult for them to engage with stories. In fact, 86% (141/164) of the mothers stated that they would recommend this DST intervention to their relatives, friends, or colleagues, suggesting the high acceptability of the intervention.

The findings suggest that the DST intervention changed Korean American and Vietnamese American mothers’ attitudes toward HPV vaccination. Health care providers’ recommendations are one of the most potent factors for promoting HPV vaccination [36,37]; however, only 30.5% (50/164) of our sample learned about HPV vaccines from health care providers. It may be that language barriers further hinder communication between first-generation immigrants and their providers. This finding suggests the need for health care providers to make special efforts with Korean American and Vietnamese American mothers to advocate and make recommendations for HPV vaccination in their practices, in addition to culturally based messaging through methods like our DST intervention.

Although prior research [19,20] using DST interventions to promote HPV vaccination yielded encouraging results, they targeted highly acculturated female Korean American college students and Khmer mother-daughter dyads. It has been noted that conducting intervention studies with first-generation immigrant populations is challenging due to language and cultural barriers and time constraints. Thus, there is a need to deliver effective interventions using technology that can overcome those challenges. The promising findings from our study suggest that the unique barriers of language and culture for the Korean American and Vietnamese American populations may be mitigated by a remotely delivered, culturally aligned DST intervention that describes scenarios for accessing health information and care using voices from their community in an easily delivered and highly accessible digital video format. Future studies need to test the use of secure platforms for enhancing intervention delivery by, for example, controlling and tracking the exposure to videos throughout the study, and allowing participants to view videos and complete surveys via a user-friendly interface from any location with internet access.

Previous studies have indicated that the child’s sex may play a role in HPV vaccination attitudes and intentions among Asian American parents [36,38]. Men are significantly less likely than women to vaccinate against this virus as the vaccine was often marked as cervical cancer prevention for women [39]. Furthermore, most research targeting Korean Americans and Vietnamese Americans has addressed HPV vaccination issues in women only [7,11,15,19]. In this study, however, the findings suggested that the relationship between the mother’s attitudes and the vaccination intention did not differ by the target child’s sex or ethnicity. Our digital stories in both Korean American and Vietnamese American groups revealed several perceived barriers and overcoming strategies as an immigrant (eg, language barriers, the complexity of the health care system, and competing family values) and reasons for choosing vaccination (eg, protection for sons and daughters and cancer prevention). It is possible that participants may be linked to the unique cultural view of the HPV attitudes, intentions, and behaviors to promote vaccination as a first-generation immigrant regardless of the child’s sex and ethnicity.

Despite the importance of disaggregating diverse Asian American subgroups and including Asian American men in this line of research, this literature has not addressed these gaps adequately. Findings from this study contribute to the knowledge about the preliminary effectiveness of using a DST intervention to promote adolescent boys’ and girls’ HPV vaccination in 2 separate at-risk and understudied Asian American or immigrant populations. Although the findings did not suggest a moderating effect of a child’s sex and ethnicity between the relationship of mothers’ attitudes and intention to vaccinate their children, it is important to note that HPV infection and related cancers affect both men and women, and the vaccine is recommended for all sexes. Additional research that examines the interrelationships among children’s sex, vaccine intention, vaccine uptake, and mothers’ ethnicity in different generations of immigrants, nonimmigrants, or other Asian American subgroups is needed. Future research with larger and more diverse samples, using rigorous designs (eg, RCTs) that incorporate objective assessments of the child’s postintervention vaccination status would better demonstrate the true effectiveness of the intervention and allow for examination of factors that contribute to (eg, either mediate or potentiate) intervention effects.

### Limitations

While this study provides feasibility, acceptability, and preliminary support for the effect of a DST intervention on vaccination intention among Korean American and Vietnamese American mothers, it is not without limitations. The modest sample size and lack of longitudinal assessments in this pilot study limit the inferences that can be drawn about the DST intervention efficacy in this study. Likewise, without a control group, we could not examine how the observed change in reported vaccination intention might have differed from changes observed in a nonintervention group. As we are still collecting long-term follow-up data regarding vaccine uptake, the effect of the intervention on the HPV vaccine uptake is not included. Finally, the sample was recruited through convenience sampling in communities. Therefore, our findings may not be broadly generalized beyond Korean American and Vietnamese American mothers. Future research could benefit from suggested directions and important design features mentioned earlier to address the limitations. Despite the limitations, our innovative culturally and linguistically congruent DST intervention showed high potential for promoting Korean American and Vietnamese American mothers’ intention to vaccinate their children. Our findings also add meaningful practical and research contributions to HPV-related cancers, a highly significant public health issue that can be prevented by timely vaccination among 2 vulnerable and underserved Asian immigrant populations.

### Conclusions

The number of Asian Americans in the United States has nearly doubled between 2000 and 2019 [40]. Korean Americans and Vietnamese Americans and Korean and Vietnamese immigrants are, respectively, the fourth and fifth largest Asian American populations and immigrant populations in the United States [40]. The current HPV vaccination rate among youth has not reached the 80% HPV vaccination rate targeted by Health People 2030 [12], with clear disparities between our target populations and other groups. Our culturally and linguistically congruent DST intervention demonstrates the high potential to reach these at-risk populations and shows promising results to increase Korean American and Vietnamese American mothers’ intention to vaccinate their children against HPV-related cancers. Importantly, this low-cost DST intervention is easy and feasible to deliver and can be scaled quickly. Future research using an RCT with a longitudinal design and powered samples that targets youth vaccine uptake as an outcome is warranted to inform the efficacy and effectiveness of the intervention.

## Abbreviations

DST: digital storytelling
HPV: human papillomavirus
OR: odds ratio
RCT: randomized controlled trial

## Data Availability

The data sets generated and analyzed during this study will be available from the first 2 authors on reasonable request.

## References

[1] (2023). HPV infection. US Centers for Disease Control and Prevention.

[2] (2022). Cancers associated with human papillomavirus (HPV). US Centers for Disease Control and Prevention.

[3] Priyadarshini M, Prabhu VS, Snedecor SJ, Corman S, Kuter BJ, Nwankwo C, Chirovsky D, Myers E (2020). Economic value of lost productivity attributable to human papillomavirus cancer mortality in the United States. Front Public Health.

[4] (2016). CDC recommends only two HPV shots for younger adolescents. US Centers for Disease Control and Prevention.

[5] Liao CI, Francoeur AA, Kapp DS, Caesar MAP, Huh WK, Chan JK (2022). Trends in human papillomavirus-associated cancers, demographic characteristics, and vaccinations in the US, 2001-2017. JAMA Netw Open.

[6] Bates JH, Hofer BM, Parikh-Patel A (2008). Cervical cancer incidence, mortality, and survival among Asian subgroups in California, 1990-2004. Cancer.

[7] Yi JK, Anderson KO, Le YC, Escobar-Chaves SL, Reyes-Gibby CC (2013). English proficiency, knowledge, and receipt of HPV vaccine in Vietnamese-American women. J Community Health.

[8] (2014). Cervical cancer is preventable. US Centers for Disease Control and Prevention.

[9] Bastani R, Glenn BA, Tsui J, Chang LC, Marchand EJ, Taylor VM, Singhal R (2011). Understanding suboptimal human papillomavirus vaccine uptake among ethnic minority girls. Cancer Epidemiol Biomarkers Prev.

[10] Nomura K, Rahman M (2014). HPV vaccine uptake among Asian American girls aged 9-17 years during 2008-2010. Int J Gynaecol Obstet.

[11] Kim B, Kim J, Kim S, Kim DS (2016). What is the awareness, perception, and rate of HPV vaccination and Pap screening amongst Korean American women?. Obstet Gynecol.

[12] (2023). Increase the proportion of adolescents who get recommended doses of the HPV vaccine—IID-08. U.S. Department of Health and Human Services, Office of Disease Prevention and Health Promotion.

[13] Hirth J (2019). Disparities in HPV vaccination rates and HPV prevalence in the United States: a review of the literature. Hum Vaccin Immunother.

[14] Kim D, Hong C, Kim J, Valdez A (2015). Improving human papillomavirus vaccine awareness and education in the Korean American community. Obstet Gynecol.

[15] Kim K, Kim B, Choi E, Song Y, Han HR (2015). Knowledge, perceptions, and decision making about human papillomavirus vaccination among Korean American women: a focus group study. Womens Health Issues.

[16] Cha C, Kim E (2013). Assessing the role of culture in Korean goose mothers' lives. J Transcult Nurs.

[17] Chen ACC, Kim WS, Larkey L (2019). Developing and pilot testing a digital storytelling intervention to promote HPV vaccination among Vietnamese American adolescents. J Nurs Healthc.

[18] (2021). Frequently Asked Questions (FAQs) about foreign born. United States Census Bureau.

[19] Kim M, Lee H, Kiang P, Aronowitz T, Sheldon LK, Shi L, Allison JJ (2020). A storytelling intervention in a mobile, web-based platform: a pilot randomized controlled trial to evaluate the preliminary effectiveness to promote human papillomavirus vaccination in Korean American college women. Health Educ Behav.

[20] Lee H, Kim M, Cooley ME, Kiang PNC, Kim D, Tang S, Shi L, Thiem L, Kan P, Peou S, Touch C, Chea P, Allison J (2018). Using narrative intervention for HPV vaccine behavior change among Khmer mothers and daughters: A pilot RCT to examine feasibility, acceptability, and preliminary effectiveness. Appl Nurs Res.

[21] Gubrium A (2009). Digital storytelling: an emergent method for health promotion research and practice. Health Promot Pract.

[22] Cumming Grant P, Currie Heather D, Moncur Rik, Lee Amanda J (2010). Web-based survey on the effect of digital storytelling on empowering women to seek help for urogenital atrophy. Menopause Int.

[23] Cueva M, Kuhnley R, Revels LJ, Cueva K, Dignan M, Lanier AP (2013). Bridging storytelling traditions with digital technology. Int J Circumpolar Health.

[24] Kim SW, Chen ACC, Ou L, Larkey L, Todd M, Han Y (2023). Developing a culturally and linguistically congruent digital storytelling intervention in Vietnamese and Korean American mothers of human papillomavirus-vaccinated children: feasibility and acceptability study. JMIR Form Res.

[25] Larkey LK, Hecht M (2010). A model of effects of narrative as culture-centric health promotion. J Health Commun.

[26] Whitehead AL, Julious SA, Cooper CL, Campbell MJ (2016). Estimating the sample size for a pilot randomised trial to minimise the overall trial sample size for the external pilot and main trial for a continuous outcome variable. Stat Methods Med Res.

[27] Teare MD, Dimairo M, Shephard N, Hayman A, Whitehead A, Walters SJ (2014). Sample size requirements to estimate key design parameters from external pilot randomised controlled trials: a simulation study. Trials.

[28] Harris PA, Taylor R, Thielke R, Payne J, Gonzalez N, Conde JG (2009). Research electronic data capture (REDCap)—a metadata-driven methodology and workflow process for providing translational research informatics support. J Biomed Inform.

[29] (2016). Guidelines for translating CAHPS® surveys. Agency for Healthcare Research and Quality.

[30] Chen ACC, Kim WS, Todd M, Larkey L (2023). Promoting Vietnamese American youths' HPV vaccination through digital storytelling.

[31] Lohr AM, Raygoza Tapia Jhenitza P, Valdez ES, Hassett LC, Gubrium AC, Fiddian-Green A, Larkey L, Sia IG, Wieland ML (2022). The use of digital stories as a health promotion intervention: a scoping review. BMC Public Health.

[32] Hopfer S (2012). Effects of a narrative HPV vaccination intervention aimed at reaching college women: a randomized controlled trial. Prev Sci.

[33] Larkey LK, Gonzalez J (2007). Storytelling for promoting colorectal cancer prevention and early detection among Latinos. Patient Educ Couns.

[34] Larkey LK, Lopez AM, Minnal A, Gonzalez J (2009). Storytelling for promoting colorectal cancer screening among underserved Latina women: a randomized pilot study. Cancer Control.

[35] Larkey L, Del Toro-Mejías Lizbeth, DiFulvio G, Gubrium A (2018). Narrative influences on "desire to act in my community" in digital storytelling workshops for Latina teens. Int Q Community Health Educ.

[36] Vu M, Berg CJ, Escoffery C, Jang HM, Nguyen TT, Travis L, Bednarczyk RA (2020). A systematic review of practice-, provider-, and patient-level determinants impacting Asian-Americans' human papillomavirus vaccine intention and uptake. Vaccine.

[37] Dempsey AF, O'Leary ST (2018). Human papillomavirus vaccination: narrative review of studies on how providers' vaccine communication affects attitudes and uptake. Acad Pediatr.

[38] Zhu L, Zhai S, Siu PT, Xia HY, Lai S, Zambrano CN, Ma GX (2019). Factors related to Chinese parents' HPV vaccination intention for children. Am J Health Behav.

[39] Boersma P, Black LI (2020). Human papillomavirus vaccination among adults aged 18-26, 2013-2018. NCHS Data Brief.

[40] (2021). Key facts about Asian Americans, a diverse and growing population. Pew Research Center.

